# Gender Disparities in Hypertensive Emergency Admissions: A National Retrospective Cohort Study

**DOI:** 10.7759/cureus.40287

**Published:** 2023-06-12

**Authors:** Garry Francis-Morel, Nehemias A Guevara, Mushrin Malik, David Sotello

**Affiliations:** 1 Internal Medicine, St. Barnabas Hospital Health System, Bronx, USA; 2 CoxHealth Pulmonology, CoxHealth Pulmonology and Sleep Medicine, Branson, USA

**Keywords:** gender disparity, hypertensive emergency, mortality, hypertensive crisis, hypertension

## Abstract

Background

Hypertension is one of the most common conditions affecting almost one in every five adults globally and hypertensive emergency is a life-threatening complication of uncontrolled hypertension leading to significant disability. Despite advances in treatment, gender disparities are yet to be addressed.

Methods

This retrospective cohort study used nationally representative data from the Healthcare Cost and Utilization Project (HCUP), specifically the National Inpatient Sample, to study two cohorts divided by sex (males and females). The primary outcome was all-cause inpatient mortality. Multivariate logistic regression analysis yielded adjusted odds ratios (aORs) for confounders. Secondary outcomes included length of stay (LOS) and total hospital charges. Multivariate linear regression identified independent predictors. We described crude rates of mechanical ventilation, acute kidney injury (AKI) requiring hemodialysis (HD), and vasopressor requirements. Patient demographics were also presented. We used the chi-squared (χ^2^) test for categorical variables and Student's t-test for continuous variables. Statistical significance was defined as a two-tailed p-value<0.05.

Results

A total of 229,025 patients met the inclusion criteria, where 52% were male and 48% were female. The mean patient age was 58 years (55 for men and 62 for women, p <0.001). White patients represented 40% of hospitalizations (males: 37%; females: 42%), black patients represented 42% (males: 43%; females: 41%), and Hispanics 11% (males: 12%; females: 10%). Medicare was the primary payer 47% of the time (males: 38%; females: 56%), Medicaid in 21% (males: 23%; females: 18%), private insurance in 20% (males: 23%; females: 17%), and no insurance in 10% (males: 14%; females: 7%). Female patients had higher rates of chronic obstructive pulmonary disease (COPD) (21% for females vs. 15% for males), connective tissue disease (4.6% for females vs. 0.98% for males; p<0.001), and dementia (6% for females vs. 3% for males). Conversely, males had a higher rate of chronic kidney disease (CKD) (51% vs. 42% for females). Male sex was a predictor of mortality (aOR 1.39, p=0.036), along with age (aOR 1.02, p<0.001) and Charlson Comorbidity Index (http://mchp-appserv.cpe.umanitoba.ca/viewConcept.php?printer=Y&conceptID=1098) (aOR 1.20, p<0.001). Sex was not a predictor of length of stay (LOS) (p=0.496) or total hospital charges (p=0.192).

Conclusions

In an attempt to achieve better outcomes in patients affected by hypertensive emergency, our retrospective cohort study found that male patients who experienced hypertensive emergency had 39% higher odds of mortality than female patients. Age and Charlson Comorbidity Index were additionally found to be independent predictors of mortality.

## Introduction

According to the World Health Organization (WHO), hypertension affects 1.28 billion people globally, with 46% of them unaware of their condition, and only one in five people with hypertension having their blood pressure under control [1]. In the United States, the Center for Disease Control and Prevention (CDC) defines hypertension as having a systolic blood pressure greater than 130 mmHg or diastolic blood pressure greater than 80 mmHg. According to their estimates, 47% of the adult population in the United States has hypertension [2].

A hypertensive emergency is a serious complication that can arise due to prolonged and uncontrolled hypertension, triggered by non-adherence to prescribed treatment leading to an abrupt surge in blood pressure, resulting in acute damage to vital organs which may lead to long-term disability or chronic morbidity. Hypertensive emergencies encompass severe conditions, such as hemorrhagic stroke, ischemic stroke, myocardial infarction, acute heart failure, and other debilitating diseases requiring immediate medical attention for proper management and care-taking measures. The consequences stemming from hypertensive emergencies are significant acutely and have substantial long-term ramifications on patients' well-being and quality of life if left untreated or inadequately managed over time [3-5].

The risk factors for hypertensive emergencies include being Caucasian, older, and having a sedentary lifestyle, among others [6]. Currently, there is limited knowledge regarding the potential gender differences in patients diagnosed with a hypertensive emergency, and further academic exploration and research are required to gain a more comprehensive understanding of this topic. The available literature on hypertensive emergency patients falls short of fully exploring potential gender disparities. Therefore, there is a pressing need for more rigorous academic inquiry and empirical research to investigate this subject matter comprehensively. Despite numerous epidemiological studies, there is a lack of research examining the impact of sex on outcomes, with conflicting data on whether male patients have a higher mortality rate.

Therefore, our study aimed to address this gap and investigate gender disparities in-hospital mortality. We also examined whether sex affected hospital length of stay, total charges, the need for hemodialysis and mechanical ventilation, and the prevalence of common comorbidities.

## Materials and methods

Data source

This retrospective cohort study utilized the National Inpatient Database, which is the largest publicly available inpatient database in the United States. The database has been available since 1988 as part of the Healthcare Cost and Utilization Project (HCUP) sponsored by the Agency for Healthcare Research and Quality (AHRQ). It includes data from 48 states plus the District of Columbia, comprising over seven million in-hospital stays annually. Long-term acute-care hospitals and rehabilitation centers were excluded from the study, and data from five years (2016-2020) were obtained. The study examined several variables, including age, sex, race, and length of stay. Diagnoses were coded using the International Classification of Diseases, 10th revision (ICD-10). The study did not include individually identifiable health information, and discharge weights were provided to facilitate the production of national estimates. Therefore, all values presented in the study are weighted estimates. The study adhered to data use agreement regulations following completed training to ensure patient privacy and confidentiality. Overall, the use of the National Inpatient Database provided a robust and comprehensive source of data for our study, allowing for the examination of large-scale trends and outcomes in patients with various medical conditions.

Study population

This study utilized billable codes from the ICD-10 to identify patients admitted for hypertensive emergency from January 2016 to December 2020. The study was limited to non-elective admissions of patients aged 18 years and older, and younger patients were excluded. Patient and hospital characteristics were identified using the National Inpatient Database (NIS) variables. To comply with the HCUP data use agreement, variables with less than 10 observations were excluded from the analysis. This study utilized a rigorous approach to identify and include only relevant patient populations in the analysis, ensuring the accuracy and validity of the results.

Missing data

In this study, missing data on variables of interest, including race, death, elective admission, female sex, insurance, total charges, and income, were observed in a small percentage of cases, with rates ranging from 0.01% to 3.4% in 2020. These rates were comparable to those of previous years, except for race in 2016, which had a higher rate of missing data (5.4%). To maintain data integrity and ensure the accuracy of the results, hospitalizations with missing data were excluded from the analysis. The study employed a rigorous approach to address missing data, minimizing its impact on the analysis and conclusions.

Study outcomes

In this study, our primary outcome was mortality, which we defined as in-hospital mortality during admission. Our secondary outcomes were the length of stay (LOS) and total hospital cost, defined as the number of days from admission to discharge or death and the total cost of hospitalization in USD, respectively. Additionally, we described the prevalence of common comorbidities and requirements for mechanical ventilation (MV), continuous renal replacement therapy (CRRT), and acute kidney injury requiring hemodialysis. We divided the studied group into two following cohorts based on gender: males and females. 

Statistical analysis

The statistical analysis of the data collected in this study was performed using the STATA MP/17 version (College Station, TX: StataCorp). Initially, we identified the number of patients who fulfilled the inclusion criteria and recorded their baseline demographic and hospital characteristics. The primary outcome of this study was all-cause mortality, which we analyzed using multivariate logistic regression to adjust for confounding variables. The secondary outcomes of the study were the length of stay (LOS) and total hospital charges, which we analyzed using multivariate linear regression. We employed the chi-squared test to assess categorical variables, while continuous variables were analyzed using Student's t-test. Statistical significance was determined as a two-tailed P-value of less than 0.05.

Patient confidentiality and institutional board review

The HCUP databases consist of limited datasets, which are healthcare data from which 16 direct identifiers have been removed in accordance with the privacy rule. Based on the HIPAA privacy rule at 45 CFR (https://www.ecfr.gov/current/title-45), limited datasets are exempt from Institutional Review Board (IRB) oversight. The present study adhered to the principles outlined in the World Medical Association (WMA) Declaration of Helsinki (https://www.wma.net/policies-post/wma-declaration-of-helsinki-ethical-principles-for-medical-research-involving-human-subjects/) regarding the ethical conduct of research involving human subjects, particularly with respect to patient confidentiality.

## Results

Patient characteristics

The study sample was composed of 229,025 admissions that were identified over five years. A total of 120,326 males (52.51%) and 108,698 females (47.49%), with a mean age of 58 years were present (males 55 vs. females 62, p<0.001). The patient population was predominantly White (40.1%), followed by Black (42.57%), Hispanic (11.3%), Asian/PI (2.63%), and Native American (0.52%); differences between males and females were statistically significant (p<0.001) but not substantial enough to draw conclusions. The majority of patients were insured by Medicare, accounting for 47.72% of the total, followed by Medicaid (21.06%), private insurance (20.39%), and no insurance (10.82%). Statistically significant differences were observed between males and females (p<0.001), with females having a higher percentage of Medicare coverage and males having a higher percentage of private insurance. Regionally, the study population was evenly distributed across the Midwest (20.86%), Northeast (16.22%), South (47.4%), and West (15.52%). The Charlson Comorbidity Index (CCI) was used to measure comorbidity, with a CCI score of 0 observed in 13.85% of the sample, while a CCI score of 3 or greater was seen in 50.64% of patients. Differences between males and females were statistically significant (p<0.001), with females having a higher percentage of CCI scores greater than or equal to 3. The mean hospital LOS was 3.5 days (p=0.467), and the mean total hospital charges were $42,247 (p=0.002). Table 1 shows the patient characteristics.

**Table 1:** Characteristics of patients included in the study. LOS: length of stay; PI: Pacific Islander

Variables		Total	Males (n=120,326)	Females (n=108,698)	p- Value
Total		229,025	52.51	47.49	NA
Age (mean in years)		58	55	62	<0.001
Race	White	40.1	37.67	42.3	<0.001
	Black	42.57	43.93	41.34	
	Hispanic	11.3	12.31	10.38	
	Asian/PI	2.63	2.4	2.83	
	Native American	0.52	0.56	0.48	
	Other	2.89	3.13	2.68	
Median household income	$1-$49,999	42.58	42.38	42.76	0.146
	$50,000-$64,999	25.35	25.85	24.89	
	$65,000-85,999	19.75	19.59	19.89	
	$86,000 or more	12.33	12.18	12.46	
Insurance	Medicare	47.72	38.32	56	<0.001
	Medicaid	21.06	23.55	18.87	
	Private	20.39	23.47	17.68	
	No insurance	10.82	14.65	7.45	
Region of hospital	Northeast	16.22	16.44	16.02	0.001
	Midwest	20.86	20.73	20.98	
	South	47.4	46.69	48.05	
	West	15.52	16.14	14.95	
Relative bed size category of hospital	Small	20.18	20.3	20.07	0.836
	Medium	29.55	29.51	29.59	
	Large	50.27	50.19	50.34	
Ownership of hospital	Government, non-federal	13.86	14.96	12.86	<0.001
	Private, non-profit	70.52	69.82	71.15	
	Private, investor-own	15.62	15.22	15.99	
Location/teaching status of hospital	Rural	6.72	6.32	7.07	<0.001
	Urban non-teaching	17.51	16.79	18.17	
	Urban teaching	75.77	76.89	74.76	
Charlson Comorbidity Index (CCI)	CCI 0	13.85	13.25	14.4	<0.001
	CCI 1	17.04	16.01	17.96	
	CCI 2	18.47	18.73	18.23	
	CCI≥3	50.64	52	49.41	
Mean hospital LOS (days)		3.5	3.5	3.6	0.467
Mean total hospital charges		42,247	42,962	41,599	0.002
Died		0.38 (n=865)	0.4	0.36	0.456

Associated conditions and complications during hospitalizations

Overall, there are several notable differences between males and females regarding the prevalence of certain conditions and complications during hospitalizations. For example, females had a significantly higher prevalence of connective tissue disease, diabetes with and without complications, dementia, cerebrovascular accident, and chronic obstructive pulmonary disease (COPD). On the other hand, males had a significantly higher prevalence of metastatic cancer and AIDS. It is also worth noting that there were several conditions where there were no significant differences between males and females. These include mechanical ventilation, acute kidney injury (AKI) requiring hemodialysis, moderate-to-severe liver disease, hemiplegia, peptic ulcer, and peripheral vascular disease. Table 2 demonstrates associated conditions and complications during hospitalizations.

**Table 2:** All the clinically significant associated conditions and complications affecting the patients included in our study.

Variables	Total	Males	Females	p-Value
Mechanical ventilation	1.36	1.4	1.32	0.440
Acute kidney injury requiring hemodialysis	4.11	4.14	4.06	0.814
Liver disease (mild)	3.15	3.87	2.5	<0.001
Liver disease (moderate-to-severe)	0.24	0.27	0.21	0.236
Connective tissue disease	2.88	0.98	4.6	<0.001
Diabetes without complications	14.68	13.05	16.15	<0.001
Diabetes with complications	25.61	24.83	26.3	<0.001
Dementia	4.97	3.23	6.55	<0.001
Cerebrovascular accident	17.36	15.7	18.87	<0.001
Hemiplegia	1.44	1.48	1.41	0.536
Chronic kidney disease	46.52	51.18	42.31	<0.001
Cancer	1.56	1.41	1.7	0.010
Metastatic cancer	0.47	0.4	0.52	0.048
Acquired immune deficiency syndrome	0.49	0.59	0.4	0.003
Peptic ulcer disease	0.63	0.59	0.67	0.308
Chronic obstructive pulmonary disease	18.57	15.83	21.05	<0.001
Acute myocardial infarction	18.19	19.82	16.71	<0.001
Peripheral vascular disease	9.42	8.97	9.82	0.002

Predictors of mortality

Males had significantly higher odds of mortality than females (aOR=1.39, 95% CI=1.02-1.91, p=0.036). Age was also a significant predictor of mortality, with each additional year increasing the odds of mortality by a factor of 1.02 (95% CI=1.01-1.04, p<0.001). The Charlson Comorbidity Index was also a significant predictor of mortality, with each additional point in the index increasing the odds of mortality by a factor of 1.20 (95% CI=1.13-1.28, p<0.001). Race, location, or teaching status did not impact mortality (p>0.05). Table 3 demonstrates predictors of mortality.

**Table 3:** The predictors of mortality in patients with hypertensive emergencies and odds ratio, 95% confidence interval and the p-value are included. PI: Pacific Islander

Variables		aOR	95% Confidence interval	p-Value
Males		1.39	1.02-1.91	0.036
Age (in years)		1.02	1.01-1.04	<0.001
Race	White	Ref	Ref	Ref
	Black	1.08	0.76-1.54	0.643
	Hispanic	0.90	0.52-1.55	0.713
	Asian/PI	1.09	0.43-2.72	0.854
	Native American	1.23	0.16-9.02	0.835
	Others	1.96	0.97-3.97	0.060
	Charlson Comorbidity Index	1.20	1.13-1.28	<0.001
Location/teaching status of hospital	Rural	Ref	Ref	Ref
	Urban non-teaching	1.31	0.56-3.03	0.522
	Urban teaching	1.62	0.76-3.48	0.208

Predictors of length of stay

Age, with a coefficient of 0.004 indicates that the length of stay increases by 0.004 days for each one-year increase in age, and this effect is statistically significant (p=0.002). CCI has a coefficient of 0.427, indicating that patients with a higher Charlson Comorbidity Index score have a longer length of stay. This effect is statistically significant (p<0.001). The location/teaching status of the hospital is also a significant predictor of length of stay. Patients in urban teaching hospitals have the longest length of stay, with a coefficient of 0.509, followed by those in urban non-teaching hospitals (coefficient of 0.169). Patients in rural hospitals have the shortest length of stay (reference group). These effects are statistically significant, with p-values of <0.001 for urban teaching hospitals and 0.022 for urban non-teaching hospitals. Table 4 demonstrates predictors of length of stay.

**Table 4:** The predictors of length of stay in patients with hypertensive emergencies and coefficient, 95% confidence interval and p-value are included. PI: Pacific Islander

Variables		Coefficient	95% Confidence interval	p-Value
Males		-0.026	-0.103-0.050	0.496
Age (in years)		0.004	0.001-0.006	0.002
Race	White	Ref	Ref	Ref
	Black	0.071	-0.019-0.161	0.124
	Hispanic	-0.093	-0.218-0.030	0.140
	Asian/PI	-0.187	-0.388-0.013	0.068
	Native American	-0.315	-0.729-0.097	0.134
	Others	0.236	-0.100-0.574	0.169
	Charlson Comorbidity Index	0.427	0.404-0.451	<0.001
Location/teaching status of hospital	Rural	Ref	Ref	Ref
	Urban non-teaching	0.169	0.024-0.314	0.022
	Urban teaching	0.509	0.386-0.632	<0.001

Predictors of hospital costs

The mean total hospitalization cost was $42,247. Hispanic or Asian/Pacific Islander races, being treated at an urban non-teaching or urban teaching hospital, and having a higher Charlson Comorbidity Index score are all associated with higher healthcare costs, on average (p<0.05). Table 5 shows the predictors of cost.

**Table 5:** The predictors of total cost in patients with hypertensive emergencies and coefficient, 95% confidence interval and p-value are included. PI: Pacific Islander

Variables		Coefficient	95% Confidence interval	p-Value
Males		615	-309 to 1,540	0.192
Age (in years)		-32	-62 to -3	0.031
Race	White	Ref	Ref	Ref
	Black	-941	-2,086 to 203	0.107
	Hispanic	8,477	6,656 to 10,297	<0.001
	Asian/PI	9,213	5,956 to 12,470	<0.001
	Native American	-4,972	-11,165 to 1,220	0.116
	Others	10,040	6,149 to 13,931	<0.001
	Charlson Comorbidity Index	3,944	3,656 to 4,233	<0.001
Location/teaching status of hospital	Rural	Ref	Ref	Ref
	Urban non-teaching	13,568	11,912 to 15,224	<0.001
	Urban teaching	14,434	13,080 to 15,788	<0.001

## Discussion

Worldwide, hypertension is a major health challenge, and despite groundbreaking advancements in its management, uncontrolled hypertension is still a major cause of hospitalization in the United States of America [7]. Moreover, as a result, we witness hypertensive crises secondary to suboptimally controlled blood pressure. Hypertensive emergency and urgency are two types of hypertensive crisis. A hypertensive emergency is characterized by the presence of target organ damage, which is not a case of hypertensive urgency [8]. With hypertensive emergencies being a major cause of morbidity in the United States affecting both genders, having a better understanding of the gender disparities is the main aim of our study.

In our study, we found 120,326 males (52.51%) and 108,698 females (47.49%), with a mean age of 58 (males 55 vs. females 62 years, p <0.001). Similar results were observed in another study using National Inpatient Database (NIS), which might be explained by the higher comorbid conditions in men than women [9]. In that study, it was also observed that men admitted with hypertensive crises were younger than women, which might be due to the fact that blood pressure control worsens in women as they grow older [9], which is linked to hormone changes [10,11]. On the contrary, one study found that female patients were 2.494 times more likely to develop hypertensive emergencies than males (95% CI: 1.111‒5.596). However, in that study, more than half of the study population were females [12]. However, another study conducted also observed that the female sex was associated with increased incidences of hypertensive crisis, which might have been a result of less understanding of hypertension-related cardiovascular complications in women [13].

Male sex was a predictor of mortality (aOR 1.39, p=0.036) as per our study. One reason behind this finding is the presence of multiple comorbidities in men that make them more prone to fatal outcomes (Charlson Comorbidity Index with p<0.001). In one study, old age, male sex, history of chronic kidney disease, and proteinuria were listed as risk factors for mortality in hypertensive patients [14]. Individuals with chronic kidney disease were found to be at increased risk of all-cause mortality secondary to hypertensive emergency [14]. Furthermore, as observed in our study, males had a higher rate of CKD (51% vs. 42% for females). Additionally, one article found that the diagnosis of hypertension is more common in women [15]. However, the incidence of hypertensive emergencies is more common in men, indicating that hypertensive men are more likely to suffer from end-organ damage [15]. Moreover, as we all know, estrogen (E2) is cardioprotective (increasing angiogenesis and vasodilation and decreasing reactive oxygen species, oxidative stress, and fibrosis) and plays a crucial role in regulating vasorelaxation, vasoconstriction, and endothelial function through endothelial Nitric Oxide Synthase dependent mechanisms. This mechanism also reduces hypertrophy on the vascular wall and increases cardiac function in hypertensive rat models. Stimulating estrogen receptors have also been shown to attenuate hypertension by reducing the expression of angiotensin-converting enzyme, levels of angiotensin II, and vasoconstriction [10]. Therefore females have better outcomes despite suffering from hypertension.

Age has been proven to be a risk factor for hypertensive emergencies. For example, one study found that the most common age group afflicted is 60-80 years [6]. In other studies, the mean age was in the fifth decade of life [16-18]. We observe this finding as one of the most important mechanisms to regulate blood pressure (BP); systemic vascular resistance (SVR) is increased in the elderly resulting in elevated BP. This increase in SVR is secondary to endothelial dysfunction, neuro-hormonal dysregulation, and a reduction in renal homeostatic mechanisms due to decreased glomerular filtration rate [17,19-21]. As a result, BP control is a bigger challenge in the elderly, leading to uncontrolled HTN, hypertensive crises, and complications.

Limitations

Our study has many strengths, such as a large sample of patients, using the National Inpatient Sample, and proper confounders variables were addressed using logistic variables regression; furthermore, the first study addressed the lack of evidence in hypertensive crises in gender disparities. However, it also has limitations; diagnoses have been collected using the ICD-10 coding system; also, just diagnosis discharges are taken into consideration and analyzed; therefore, secondary diagnoses were not included making it impossible to know if secondary diagnosis played an essential role in outcomes. Moreover, the treatment used is unavailable (NIS lacked this data); due to the nature of the data used, it was impossible to decipher if the same patient had multiple hospitalizations, which could potentially skew the data.

## Conclusions

In this retrospective cohort study, utilizing national representative data over five years, it was found that males exhibited significantly higher odds of mortality than females. Furthermore, the study identified age and CCI as independent predictors of mortality. These findings have significant implications for healthcare providers and policymakers alike. Healthcare providers should be mindful of the elevated mortality risk among male patients and may need to adopt distinct treatment approaches for male and female patients. Policymakers can also utilize these findings to inform the development of targeted public health initiatives to reduce mortality rates, particularly among at-risk groups.

## References

[1] (2023). Hypertension. https://www.who.int/news-room/fact-sheets/detail/hypertension.

[2] (2023). CDC: high blood pressure - hypertension statistics and maps. https://www.cdc.gov/bloodpressure/facts.htm.

[3] Janke AT, McNaughton CD, Brody AM, Welch RD, Levy PD (2016). Trends in the incidence of hypertensive emergencies in US emergency departments from 2006 to 2013. J Am Heart Assoc.

[4] Peixoto AJ (2019). Acute severe hypertension. N Engl J Med.

[5] Astarita A, Covella M, Vallelonga F (2020). Hypertensive emergencies and urgencies in emergency departments: a systematic review and meta-analysis. J Hypertens.

[6] Vilela-Martin JF, Vaz-de-Melo RO, Kuniyoshi CH, Abdo AN, Yugar-Toledo JC (2011). Hypertensive crisis: clinical-epidemiological profile. Hypertens Res.

[7] Almas A, Ghouse A, Iftikhar AR, Khursheed M (2014). Hypertensive crisis, burden, management, and outcome at a tertiary care center in Karachi. Int J Chronic Dis.

[8] Aggarwal M, Khan IA (2006). Hypertensive crisis: hypertensive emergencies and urgencies. Cardiol Clin.

[9] Ebinger JE, Liu Y, Driver M (2022). Sex-specific temporal trends in hypertensive crisis hospitalizations in the United States. J Am Heart Assoc.

[10] Iorga A, Cunningham CM, Moazeni S, Ruffenach G, Umar S, Eghbali M (2017). The protective role of estrogen and estrogen receptors in cardiovascular disease and the controversial use of estrogen therapy. Biol Sex Differ.

[11] Nkoke C, Jingi AM, Noubiap JJ (2022). Gender differences in cardiovascular risk factors, clinical presentation, and outcome of patients admitted with a hypertensive crisis at the Buea Regional Hospital, Cameroon. Int J Hypertens.

[12] Desta DM, Wondafrash DZ, Tsadik AG, Kasahun GG, Tassew S, Gebrehiwot T, Asgedom SW (2020). Prevalence of hypertensive emergency and associated factors among hospitalized patients with hypertensive crisis: a retrospective cross-sectional study. Integr Blood Press Control.

[13] Saguner AM, Dür S, Perrig M (2010). Risk factors promoting hypertensive crises: evidence from a longitudinal study. Am J Hypertens.

[14] Couser WG, Remuzzi G, Mendis S, Tonelli M (2011). The contribution of chronic kidney disease to the global burden of major noncommunicable diseases. Kidney Int.

[15] Zampaglione B, Pascale C, Marchisio M, Cavallo-Perin P (1996). Hypertensive urgencies and emergencies. Prevalence and clinical presentation. Hypertension.

[16] Pierin AM, Flórido CF, Santos JD (2019). Hypertensive crisis: clinical characteristics of patients with hypertensive urgency, emergency and pseudocrisis at a public emergency department. Einstein (Sao Paulo).

[17] Siqueira DS, Riegel F, Crossetti MD, Tavares JP (2015). Perfil de pacientes com crise hipertensiva atendidos em um Pronto Socorro no sul do Brasil. Rev Enferm UFSM.

[18] Andrade DO, Santos SP, Pinhel MA (2017). Effects of acute blood pressure elevation on biochemical-metabolic parameters in individuals with hypertensive crisis. Clin Exp Hypertens.

[19] Dharmashankar K, Widlansky ME (2010). Vascular endothelial function and hypertension: insights and directions. Curr Hypertens Rep.

[20] Varon J (2008). Treatment of acute severe hypertension: current and newer agents. Drugs.

[21] Singh M (2011). Hypertensive crisis-pathophysiology, initial evaluation, and management. J Indian Coll Cardiol.

